# Phosphorylation of Atg31 is required for autophagy

**DOI:** 10.1007/s13238-015-0138-4

**Published:** 2015-03-17

**Authors:** Wenzhi Feng, Tong Wu, Xiaoyu Dan, Yuling Chen, Lin Li, She Chen, Di Miao, Haiteng Deng, Xinqi Gong, Li Yu

**Affiliations:** 1 PTN Program, College of Life Science, Peking University, Beijing, 100871 China; 2 State Key Laboratory of Biomembrane and Membrane Biotechnology, Tsinghua University-Peking University Joint Center for Life Sciences, School of Life Sciences, Tsinghua University, Beijing, 100084 China; 3 Center for Biomedical Analysis, Tsinghua University, Beijing, 100084 China; 4 National Institute of Biological Sciences, Beijing, 102206 China; 5 Institute for Mathematical Sciences, Renmin University of China, Beijing, 100872 China

**Keywords:** autophagy, Atg31, phosphorylation, autophagosome, pre-autophagosomal structure (PAS)

## Abstract

Autophagy is an evolutionarily conserved cellular process which degrades intracellular contents. The Atg17-Atg31-Atg29 complex plays a key role in autophagy induction by various stimuli. In yeast, autophagy occurs with autophagosome formation at a special site near the vacuole named the pre-autophagosomal structure (PAS). The Atg17-Atg31-Atg29 complex forms a scaffold for PAS organization, and recruits other autophagy-related (Atg) proteins to the PAS. Here, we show that Atg31 is a phosphorylated protein. The phosphorylation sites on Atg31 were identified by mass spectrometry. Analysis of mutants in which the phosphorylated amino acids were replaced by alanine, either individually or in various combinations, identified S174 as the functional phosphorylation site. An S174A mutant showed a similar degree of autophagy impairment as an Atg31 deletion mutant. S174 phosphorylation is required for autophagy induced by various autophagy stimuli such as nitrogen starvation and rapamycin treatment. Mass spectrometry analysis showed that S174 is phosphorylated constitutively, and expression of a phosphorylation-mimic mutant (S174D) in the Atg31 deletion strain restores autophagy. In the S174A mutant, Atg9-positive vesicles accumulate at the PAS. Thus, S174 phosphorylation is required for formation of autophagosomes, possibly by facilitating the recycling of Atg9 from the PAS. Our data demonstrate the role of phosphorylation of Atg31 in autophagy.

**Electronic supplementary material:**

The online version of this article (doi:10.1007/s13238-015-0138-4) contains supplementary material, which is available to authorized users.

## INTRODUCTION

Autophagy is an evolutionarily conserved, lysosome-based degradation pathway. During autophagy, double-membrane vesicles are formed which engulf cytosol or damaged orgenalles in a selective or non-selective manner. Autophagy plays important roles in various physiological settings, and disruption of autophagy has been shown to lead to many pathological conditions (Winslow and Rubinsztein, 2008; Hussey et al., 2009; Mizushima and Komatsu, 2011; Jiang and Mizushima, 2014; Martin et al., 2014).

In yeast, autophagy is initiated at a specific site based on a multi-protein complex named the pre-autophagosomal structure (PAS). Formation of a double-membrane structure, named the isolation membrane, is initiated at the PAS. The isolation membrane extends and surrounds cytosolic cargoes before sealing to form the completed autophagosome.

More than 30 Atg proteins involved in autophagy have been identified using *Saccharomyces cerevisiae* as a model organism since the 1990s (Tsukada and Ohsumi, 1993; Thumm et al., 1994; Harding et al., 1995). Most of those Atg proteins can be recruited to the PAS (Suzuki and Ohsumi, 2010). At the core of the PAS is a stable ternary complex of Atg17, Atg29 and Atg31 (Kabeya et al., 2009). Atg17 interacts with Atg31 and Atg29 independent of nutrient conditions. Under nutrient starvation conditions, the Tor complex is inactivited, which causes dephosphorylation of Atg13, followed by binding of dephosphorylated Atg13 to Atg1. The Atg1-Atg13 complex is then recruited to the Atg17 complex, thus activating the autophagy pathway. Atg31 was originally found as a partner of Atg17 from yeast two-hybrid assays and global mass spectrometry analysis (Kabeya et al., 2007). Atg31 has been reported to be a phosphorylated protein, but the phosphorylation site has not been identified and the function of this phosphorylation remains to be elucidated.

In this study, we demonstrate that Atg31 is constitutively phosphorylated. Mass spectrometry identified 11 phosphorylation sites in Atg31, and analysis of mutants created by alanine swapping confirmed that S174 is the functional phosphorylation site. Autophagy is impaired to a similar degree in the S174A mutant as in the Atg31 deletion mutant. S174 phosphorylation is required for autophagy induced by nitrogen starvation, amino acid starvation and rapamycin treatment. Expression of a phosphorylation-mimic mutant (S174D) in the Atg31 deletion strain restores autophagy. Finally, we show that S174 phosphorylation is required for recycling of Atg9 from the PAS. Our data demonstrate the role of phosphorylation of Atg31 in autophagy.

## RESULTS

### Atg31 is a phosphorylated protein

**Figure 1:**
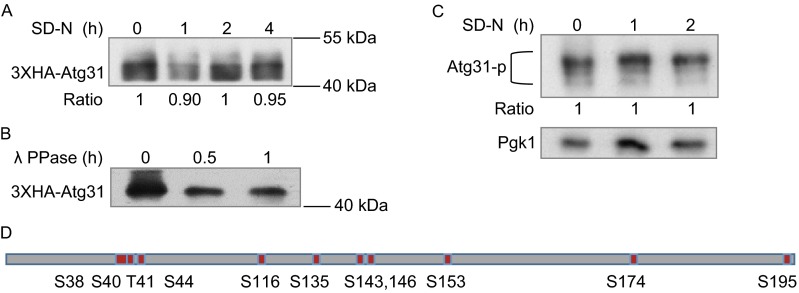
**Identification and analysis of phosphorylation sites on Atg31**. (A) Yeast cells expressing 3XHA-Atg31 were transferred to SD-N medium for 1 h, 2 h or 4 h. Cell lysates were assessed by Western blotting with HA antibody. the grey value ratio of sample 1 h, 2 h, 4 h compared to 0 h is shown. (B) Cell lysates under full (SD) medium were treated with lambda PPase at 30°C for 0 h, 0.5 h and 1 h, then samples were assessed by Western blotting with HA antibody. (C) Yeast cells expressing 3XHA-Atg31 were transferred to SD-N medium for 2 h. Cells lysates were assessed by Phos-tag Western blotting to detect phosphorylation. The ratio is the same as (A). (D) Atg31 protein was isolated by GST-tag purification from yeast grown in full or SD-N medium and analyzed by mass spectrometry. Atg31 phosphorylation sites are shown as red rectangles

We noticed that when cells were grown in both nutrient-rich and starvation conditions, the Atg31 protein displayed multiple bands of higher molecular weight when analyzed by SDS-PAGE (Fig. 1A). Thus, Atg31 appears to undergo some sort of post-translational modification in a nutrient-independent manner. Treating the cell lysate with λ phosphatase elimnated the multiple upper bands, suggesting that Atg31 is modified by phosphorylation (Fig. 1B). To better monitor the phosphorylation level of Atg31 during starvation, we used a phos-tag detection assay which enhances the mobility shifts of phosphorylated proteins on SDS-PAGE (Kinoshita et al., 2004). We found the phosphorylation level of Atg13 is similar in starved and un-starved cells (Fig. 1C).

### Identification of Atg31 phosphorylation sites

To identify the Atg31 phosphorylation sites, we tagged Atg31 with an N-terminal GST tag and purified it from yeast under nutrient-rich conditions and starvation conditions. When we analyzed the protein by mass spectrometry (MS), we identified 11 phosphorylation sites (Fig. 1D).

### Screening of functional phosphorylation sites in Atg31

**Figure 2:**
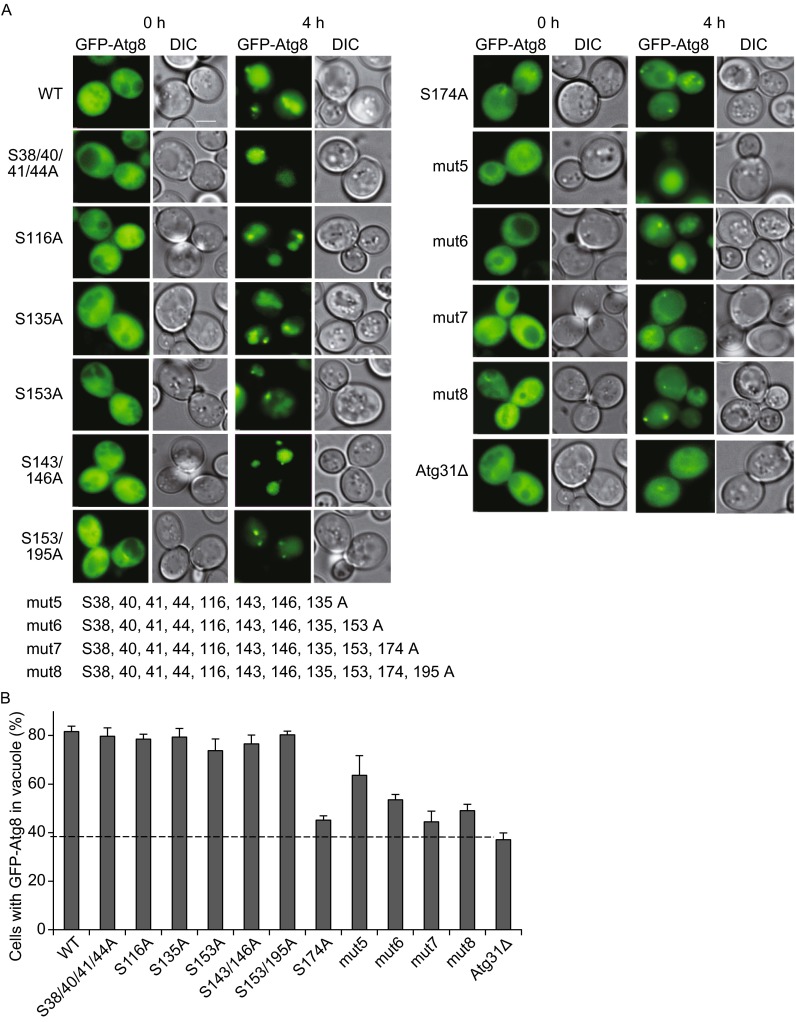
**Mutagenisis screen**. (A) The phosphorylated threonine and serine residues shown in Fig. 1D were mutated to alanine, and Atg31 mutants with single mutations or multiple mutations in various combinations, as well as Atg31 deletion mutants, were assessed for autophagy activity under nitrogen starvation conditions. Scale bar, 2 µm. (B) Autophagy activity was assessed by monitoring the translocation of GFP-Atg8 into vacuoles. 100 cells were assessed in a blind fashion and quantified. Error bars indicate standard deviation (s.d.) (*n* = 3)

Next, we screened the phosphorylation sites for their effect on autophagosome formation using GFP-Atg8 as a marker. We mutated each amino acid individually to alanine, and we also generated mutants in which various combinations of phosphorylation sites were changed to alanine. Then we assessed autophagy activity by monitoring the ability of each mutant to transport GFP-Atg8 into the vacuole. In yeast carrying the S174A single mutant, a lower percentage of cells had vacuolar Atg8. Therefore, our screening method identified the Serine at 174 mutant as a potential functional phosphorylation site (Fig. 2A and 2B).

### Phosphorylation at S174 is required for autophagy induced by various cues

**Figure 3:**
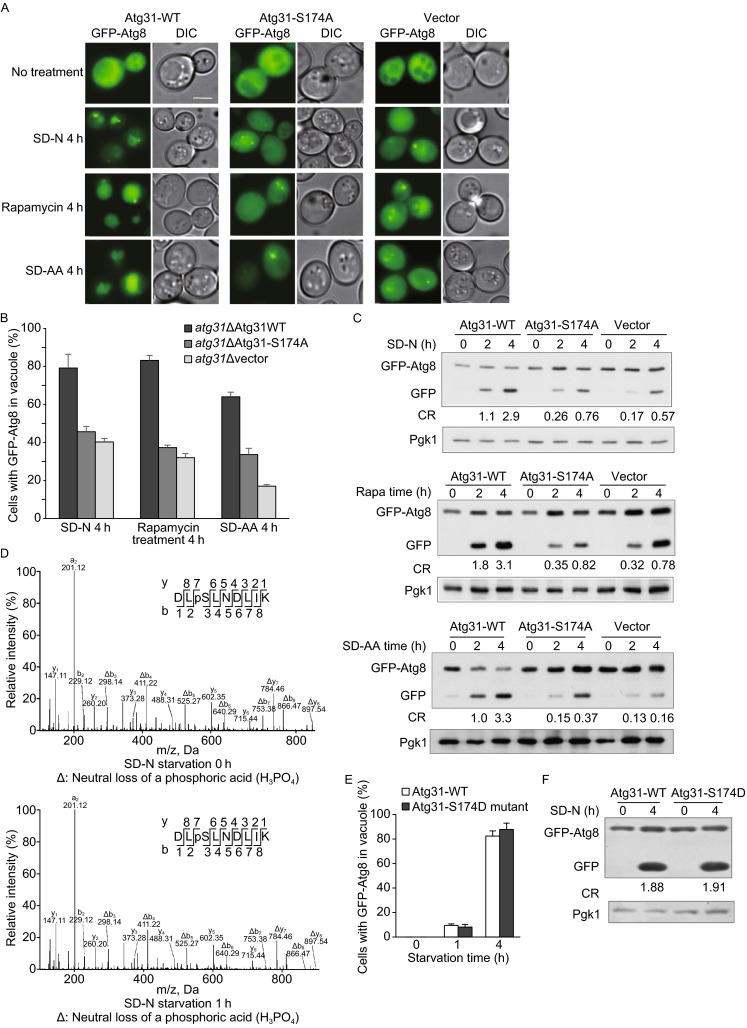
**Ser174 phosphorylation is required for autophagy under various conditions**. (A) Cells of the Atg31∆ strain expressing wild-type (WT) Atg31, the Atg31-Ser174 mutant (S174A), or a control vector were treated by nitrogen starvation (SD-N), rapamycin or amino acid starvation (SD-AA) and assessed for autophagy activity. Scale bar, 2 μm. (B) Cells from (A) were assessed for autophagy activity by monitoring the translocation of GFP-Atg8 into vacuoles. 100 cells were assessed in a blind fashion and quantified. Error bars indicate standard deviation (s.d.) (*n* = 3). (C) Cells from (A) were analyzed for GFP-Atg8 cleavage by Western blotting with GFP antibody. CR means the cleavage ratio between GFP-Atg8 and GFP, using grey value statistic analysis of three independent experiments. (D) Mass spectrometry analysis of Ser174 phosphorylation on Atg31 from cells grown in full medium (0 h) or SD-N medium (1 h). (E) The phosphomimic mutant S174D was assessed for autophagy activity by monitoring the translocation of GFP-Atg8 into vacuoles. 100 cells were assessed in a blind fashion and quantified. Error bars indicate standard deviation (s.d.) (*n* = 3). (F) Cells from (E) were analyzed for GFP-Atg8 cleavage by Western blotting with GFP antibody

To confirm the role of S174 in nitrogen starvation-induced autophagy, we compared the autophagy activity in wild-type cells and cells carrying the Atg31 S174A mutant using various stimuli including nitrogen starvation, amino acid starvation and rapamycin treatment. Autophagy activity was monitored microscopically by translocation of GFP-Atg8 into vacuoles (Fig. 3A), and biochemically by cleavage of GFP-Atg8 as detected by Western blotting (Fig. 3C). Both assays showed that autophagy activity was reduced by about 60% (Fig. 3B and 3C). It is worth noting that this reduction is similar to that observed when ATG31 deleted. Thus, we concluded that phosphorylation at S174 is essential for Atg31 to carry out its function in autophagy. Phosphorylation of S174 can be detected in cells growing in nutrient-rich conditions and in cells undergoing starvation, which implies that phosphorylation of S174 is not regulated by nitrogen starvation (Fig. 3D).

### A phosphomimic mutant rescues autophagy

To rule out the possibility that reduction of autophagy activity is due to the serine-to-alanine change, rather than loss of phosphorylation at S174, we generated a phosphomimic mutant strain in which serine 174 is replaced by aspartic acid (S174D). We found that expression of the S174D plasmid can rescue autophagy in the Atg31 deletion mutant (Fig. 3E and 3F). Thus, phosphorylation at S174 is required for autophagy.

### Impairment of Atg9 recycling in the S174A mutant

**Figure 4:**
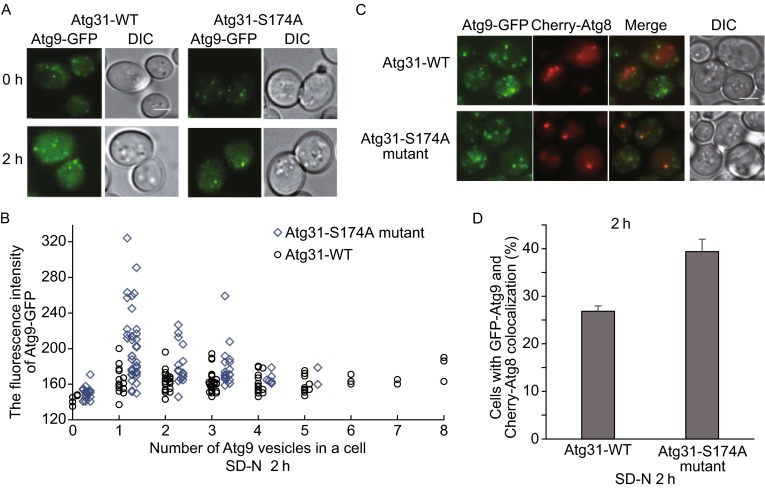
**Impairment of Atg9 recycling in the S174A mutant**. (A) Wild-type or S174A mutant cells expressing Atg9-GFP were transferred to nitrogen starvation for 0 h or 2 h and observed by confocal microscopy. Scale bar, 2 µm. (B) Cells from (A) were assessed for the number and intensity of Atg9 puncta. (C) Wild-type or S174 mutant cells expressing Atg9-GFP and Cherry red-Atg8 were transferred to SD-N medium for 2 h and observed by confocal microscopy. Scale bar, 2 µm. (D) Cells from (C) were assessed for co-localization between Atg9 and Atg8. 100 cells were assessed in a blind fashion and quantified. Error bars indicate standard deviation (s.d.) (*n* = 3)

Atg9 is a multi-pass transmembrane protein that plays a key role in autophagosome formation. Atg9-positive vesicles are highly mobile structures in the cytoplasm (Yamamoto et al., 2012) that recycle Atg9 and other molecular from the PAS to the cytoplasmic pool (Reggiori et al., 2004). We found that the number of Atg9 puncta is reduced in the S174A mutant. Furthermore, the fluorescence intensity of the Atg9 puncta is dramatically enhanced, indicating that the dynamic recycling of Atg9 between different pools is impaired and Atg9 accumulates in these puncta (Fig. 4A and 4B). Since Atg9 puncta in S174A mutants are co-localized with the PAS marker Atg8 (Fig. 4C and 4D), we conclude the recycling of Atg9 between the PAS and the cytoplasmic pool is impaired.

### S174 phosphorylation affects the interface between Atg31and Atg17

**Figure 5:**
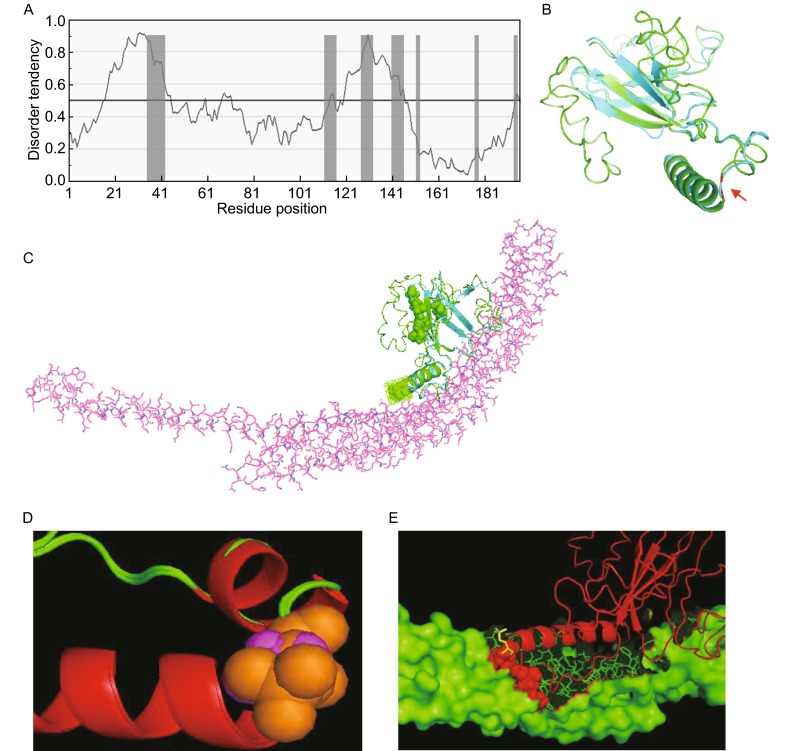
**Structure simulation of Atg31**. (A) Tendency for intrinsic disorder within the amino acid sequence of Atg31, as predicted by IUPed. The phosphorylation sites are marked by grey columns. (B) Structural modelling of Atg31. A model of Atg31 from *Saccharomyces cerevisiae* was constructed with Modeller 9.11 and superimposed on the crystallographic structure of Atg31 from the *Lachancea thermotolerans* Atg17-Atg31-Atg29 complex (Protein Data Bank code 4HPQ). The major secondary structure motifs (alpha-helixes and beta-strands) are closely aligned in the two structures. The model of *S. cerevisiae* Atg31 is in green and the X-ray-solved structure of *L. thermotolerans* Atg31 is in blue. The red arrow indicates S174. (C) Superimposition of the built model of *S. cerevisiae* Atg31 (green) on the crystallographic structure of *L. thermotolerans* Atg31 (blue) complexed with Atg17 (pink). The *L. thermotolerans* proteins are from the Atg17-Atg31-Atg29 complex (4HPQ). The phosphorylated serine residues in Atg31 are shown as spheres, and S174 is additionally indicated by a mesh. S174 is located at the interface between Atg31 and Atg17. (D) Superimposition of the local structure around S174 with or without phosphorylation. Non-phosphorylated S174 is shown as pink spheres, and phosphorylated S174 is shown as orange spheres. The loop (green) near to S174 in the unmodified structure is changed to a helix (red) by phosphorylation. (E) The arginine 171 and leucine 175 residues near the phosphorylation site are deeply buried after S174 phosphorylation. Red spheres show the two buried residues, and the yellow stick shows phospho-S174

The migration of Atg31 on SDS-PAGE is abnormal and much slower than typical globular proteins. 3XHA-tagged Atg31 shows a molecular weight of about 40–45 kDa by SDS-PAGE, and the dephosphorylated form is about 40 kDa. However, the molecular weight of Atg31 calculated from its amino acid sequence is 22 kDa. Since many proteins containing intrinsically disordered regions have similar abnormal migration on SDS-PAGE gels, we hypothesized that Atg31 may have characteristics of an intrinsically disordered protein (IDP). We used a disorder prediction tool, IUPred, to analyze Atg31. IUPred assesses the tendency of a protein to contain disordered regions based on whether the amino acid sequence allows stable interactions (Dosztanyi et al., 2005). The prediction shows that almost half of the Atg31 sequence is disordered in solution (Fig. 5A).

To elucidate the possible role of S174 phosphorylation, we built a structural model of *Saccharomyces cerevisiae* Atg31 using a homologous modeling method and a threading modeling method (Pronk et al.,^ 2013^). As shown in Fig. 5B, the *S. cerevisiae* Atg31 has a very similar structure to *Lachancea thermotolerans* Atg31 (Protein Data Bank code 4PHQ:B) (Ragusa Michael et al., 2012). After superimposing the Atg31 model structure onto the Atg17-Atg31-Atg29 complex structure (4HPQ), it is very clear that the S174 phosphorylation site is located at the interface between Atg17 and Atg31. No other phosphorylation site locates to the interface. Phosphorylation of S174 increases the number of atoms in the side chain and enlarges its volume, which will change the interaction of Atg31 with its binding partners (Fig. 5C).

Furthermore, from this model, we found that the secondary structure (SS) of the six residues 167–172 was changed from a loop to a helix by phosphorylation of S174 (Fig. 5D). The increased number of atoms in phospho-serine changes the local interactions between residue side chains and makes the flexible loop transform into a stable helix, as shown in Fig. 4D. This will also change the local motions of the C-terminal helix. S174 phosphorylation also changes the interaction between Atg31 and Atg17. Near S174, amino acids 171 (arginine) and 175(leucine) are the two most buried residues in the interface between non-phosphorylated Atg31 and Atg17. Their buried areas are 150.5 Å and 113.3 Å respectively. Phosphorylation of S174 enlarges their buried areas by 12.1 Å and 8.5 Å respectively, which results in an enlarged Atg31/Atg17 interface (Fig. 5E).

## DISCUSSION

In this study, we identified 11 phosphorylation sites on Atg31. Mutagenesis analysis showed that phosphorylation at serine 174 is required for Atg31 to carry out its function, while the other phosphorylation sites have no function in the regulation of autophagy. We further demonstrated that phosphorylation at S174 is required for efficient Atg9 recycling, and the impairment of this phosphorylation in the S174A mutant causes accumulation of Atg9 puncta and impaired autophagy.

One obvious question remains unsolved: what is the kinase responsible for phosphorylation of Atg31? So far, our efforts to identify the kinase have been hampered by the lack of a specific antibody against phospho-S174. We have failed to generate such an antibody despite repeated attempts.

Atg31 has intrinsically disordered regions which means that it can easily be phosphorylated (Tompa, 2002; Iakoucheva et al., 2004). Our structure simulations illustrate how phosphorylation at S174 changes the C-terminal loop into a helix. This helix makes additional contacts with the crescent-shaped Atg17, thus enlarging the interface between Atg31 and Atg17. In the S174A mutant, the lack of phosphorylation at S174 causes the C-terminal helix to become a flexible loop. This may result in part of the Atg17 protein becoming exposed, thus leading to abnormal PAS assembly, which eventially causes impaired Atg9 recycling.

## MATERIALS AND METHODS

### Strains and plasmids

Standard protocols were used for yeast manipulations (Kaiser, 1994). Cells were cultured at 30°C in SD medium (0.17% yeast nitrogen base without amino acids and ammonium sulfate, 0.5% ammonium sulfate, 0.5% casamino acids and 2% glucose) supplemented with appropriate nutrients. Autophagy was induced by transferring the cells to SD-N medium (0.17% yeast nitrogen base, without amino acids and ammonium sulfate, and 2% glucose). Otherwise, to induce autophagy, cells were treated with 0.2 and 0.5 μg/mL rapamycin (Sigma-Aldrich), or transferred to SD-AA medium respectively.

### Yeast strains and media

BY4741 (*MATa his3D leu2D met15Dura3D*), ScLY4 (*BY4741 atg31*::*kanMX)*, ScLY5 (*BY4741*, *ATG31*-*HA*::*HIS3)* and ScLY6 (*ScLY4*, *YEPlac181* [*Gal1*-*GST*-*ATG31*]) were used in this study. The other Atg disruptants are listed in Table S1. BY4741 was purchased from Invitrogen. Media and methods for gene disruption have been described previously (Longtine et al., 1998).

### Plasmids and other materials

The full length *atg31* gene with its endogenous promoter and terminator was amplified by PCR and ligated into pRS316, Yeplac181 and Ycplac111 plasmids with appropriate restriction endonucleases. The *atg31* ORF region and the downstream 600 bp was amplified by PCR and inserted into YEplac181 plasmid after the GAL4 promoter and the N-GST tag sequence. Site-specific mutagenesis was performed with a simple PCR method. Plasmids containing *atg31* were amplified using primers containing sequences 15 bp upstream and downstream of the mutation site. The products were cut by Dpn I (New England Biolabs) and transformed into competent *E. coli*. Mutations were confirmed by sequencing.

### Phos-tag Western blotting assay

The phos-tag assay was performed as described before with modifications (Kosako et al., 2009). For phosphate-affinity polyacrylamide gel electrophoresis, an 8%–10% separating gel containing 25 mmol/L phos-tag acrylamide (AAL-107, Wako) and 50 mmol/L MnCl_2_ was prepared with a normal stacking gel. After samples were loaded, the gel was run with a current of 15–20 mA for about 2 h. The gel was washed with transfer buffer containing 1 mmol/L EDTA to move the Mn^2+^, then proteins were transferred to PVDF membranes. The membranes were blocked, incubated with antibodies and processed according to standard procedures.

### Fluorescence microscopy

For fluorescence microscopy, cells were grown to OD_600_ = 0.8 − 1.0 in appropriate selective medium and shifted to SD-N medium for various lengths of time as described (Cheong et al., 2005). The cells were observed at room temperature using FV-1000 (Olympus) confocal microscopes. The percentages of cells with vacuolar GFP-Atg8 fluorescent signals were determined by counting 100 cells in three separate experiments (Yi et al., 2012).

### GST-tag protein purification

Plasmid Yeplac181, containing the GAL4 promoter and the ORF of the *atg31* gene, was transformed into BY4741 to purify Atg31 protein. Cells were incubated in up to 2 liters of SD-Leu medium containing 2% raffinose instead of glucose from OD_600_ = 0.2 until OD_600_ = 0.8 − 1.2 with rotation in a 30°C incubator. Galactose was added at a final concentration of 2% to induce GST-Atg31 expression for 2–4 h. One liter of the culture was harvested as non-starved cells. The remaining cells were washed three times with SD-N medium containing 2% raffinose and glactose. The cells were then starved for 1 h in this SD-N medium and then harvested and lysed together with the non-starved cells. GST-Atg31 protein was purified with Glutathione Sepharose TM 4B (GE Healthcare) as previously described (Lu et al., 2011). Cells were vortexed with glass beads to break them open, then centrifuged at high speed. The supernatant was collected and incubated with Glutathione Sepharose for 2 h. The column containing the lysate was washed slowly by wash buffer with a high NaCl concentration, and then eluted by reduced GSH (Sigma).

### Disorder prediction

The Atg31 protein sequence was used for protein disorder prediction, which was performed using online disorder prediction software, including IUPed, Pondr-FIT and ANCHOR.

### Mass spectrum analysis

For LC–MS/MS analysis, peptides were separated by a 90 min gradient elution at a flow rate of 0.250 μL/min with a Thermo-Dionex Ultimate 3000 HPLC system, which was directly interfaced with a Thermo LTQ-Orbitrap Velos pro mass spectrometer. The analytical column was a homemade fused silica capillary column (75 μm ID, 150 mm length; Upchurch, Oak Harbor, WA) packed with C-18 resin (300 A, 5 μm; Varian, Lexington, MA). Mobile phase A consisted of 0.1% formic acid, and mobile phase B consisted of 80% acetonitrile and 0.1% formic acid. An LTQ-Orbitrap mass spectrometer was operated in the data-dependent acquisition mode using Xcalibur 2.2 software and there was a single full-scan mass spectrum in the Orbitrap (400–1800 m/z, 30,000 resolution) followed by 20 data-dependent MS/MS scans in an ion trap at 35% normalized collision energy (CID).

MS/MS spectra from each LC–MS/MS run were searched against Atg31 in the *Saccharomyces cerevisiae* database using the Proteome Discoverer (Version 1.4) searching algorithm. The search criteria were as follows: full tryptic specificity was required; two missed cleavages were allowed; carbamidomethylation was set as fixed modification; oxidation (M) was set as a variable modification; precursor ion mass tolerance was 10 ppm for all MS acquired in the Orbitrap mass analyzer; and fragment ion mass tolerance was 0.8 Da for all MS2 spectra acquired in the LTQ. A high confidence score filter (FDR < 1%) was used to select the “hit” peptides and their corresponding MS/MS spectra were manually inspected.

### Computational modeling of Atg31 structure

The comparative modeling tool Modeller 9.11 was first implemented to build a model based on the homologous *Lachancea thermotolerans* Atg31 structure from the Protein Data Bank (code 4HPQ:B) (Joosten et al., 2011). In order to obtain a good model, the I-TASSER (Roy et al., 2010) server was also used, which takes advantage of multiple-threading alignments and iterative template fragment assembly simulations. The best model from Modeller and the best model from I-TASSER were picked out and minimized using the molecular dynamics simulation package Gromacs 4.5 (Gong et al., 2010) using OPLS force field^3^. Then the model with the better energy score was selected as the built model. We used Pymol^4^ to superimpose the built Atg31 model structure onto the structure of the Atg17-Atg31-Atg29 complex (Protein Data Bank code 4HPQ) (DeLano, 2002).

## Electronic supplementary material

Below is the link to the electronic supplementary material.

Supplementary material 1 (PDF 164 kb)

## ABBREVIATIONS

Atg: autophagy-related (Klionsky et al., 2003)
IDP: intrinsically disordered protein
MS: mass spectrometry
PAS: pre-autophagosomal structure
SS: secondary structure
