# Supplementary material for: Phosphorylation of Atg31 is required for autophagy
Source: Protein Cell. 2015 Mar 17;6(4):288–96. doi: 10.1007/s13238-015-0138-4 (PMC4383750; doi:10.1007/s13238-015-0138-4)
Supplement: Supplementary file 1 — Supplementary material 1 (PDF 164 kb) [file 13238_2015_138_MOESM1_ESM.pdf]

Supplemental Table1

Name	Genotype	reference
**BY4741**	*MATa his3Δ1 leu2Δ met15Δ ura3Δ*	This study
**ScLY1**	BY4741, *pRS316*[*GFP-ATG8*]	This study
**ScLY2**	BY4741, *pRS315[GFP-ATG8*]	This study
**ScLY3**	BY4741, *YEPlac181* [*Gal1-GST-ATG31*]	This study
**ScLY4**	BY4741, *atg31*Δ::*kanMX6*	This study
**ScLY5**	BY4741, *ATG31-HA*::*HIS3*	This study
**ScLY6**	ScLY4, *YEPlac181* [*Gal1-GST-ATG31*]	This study
**ScLY7**	ScLY4, *YcPlac111* [*HA-ATG31*]	This study
**ScLY8**	ScLY4, *YcPlac111* [*ATG31], pRS316*[*GFP-ATG8*]	This study
**ScLY9**	ScLY4, *YcPlac111* [*ATG31-S174A*], *pRS316*[*GFP-ATG8*]	This study
**ScLY10**	ScLY4, *YcPlac111, pRS316*[*GFP-ATG8*]	This study
**ScLY11**	ScLY4, *YEPlac181* [*ATG31*]	This study
**ScLY12**	ScLY2, *pRS416*[*ATG9-2XGFP*]	This study
**ScLY13**	ScLY4, *YcPlac111* [*ATG31*], *ATG17-GFP*::*HIS3*	This study
**ScLY14**	ScLY4, *YcPlac111* [*ATG31*], *ATG29-GFP*::*HIS3*	This study
**ScLY15**	ScLY4, *YcPlac111* [*ATG31*], *ATG1-GFP*::*HIS3*	This study
**ScLY16**	ScLY4, *ATG17-HA*::*HIS3*, *ATG1-MYC*::*natNT2*, *YcPlac111* [*ATG31*]	This study
**ScLY17**	ScLY4, *ATG17-HA*::*HIS3*, *ATG1-MYC*::*natNT2*, *YcPlac111*[*ATG31-S174A*]	This study
**ScLY18**	ScLY4, *ATG17-HA*::*HIS3*, *ATG1-MYC*::*natNT2*, *YcPlac111*	This study
**ScLY19**	ScLY4, *ATG17-HA*::*HIS3*, *ATG29-MYC*::*natNT2*, *YcPlac111* [*ATG31*]	This study
**ScLY20**	ScLY4, *ATG17-HA*::*HIS3*, *ATG29-MYC*::*natNT2*, *YcPlac111* [*ATG31-S174A*]	This study
**ScLY21**	ScLY4, *ATG17-HA*::*HIS3*, *ATG29-MYC*::*natNT2*, *YcPlac111*	This study
**ScLY22**	ScLY5, *ATG29-MYC*::*natNT2*, *pRS316*[*ATG31-HA*]	This study
**ScLY23**	ScLY4, *ATG29-MYC*::*natNT2*, *pRS316*[*ATG31-S174A-HA*]	This study
**ScLY24**	ScLY4, *ATG29-MYC*::*natNT2*, *pRS316*	This study
**ScLY25**	ScLY4, *ATG17-MYC*::*natNT2*, *pRS316*[*ATG31-HA*]	This study
**ScLY26**	ScLY4, *ATG17-MYC*::*natNT2*, *pRS316*[*ATG31-HA*]	This study
**ScLY27**	BY4741, *atg1*Δ::*kanMX6*, *YEPlac181* [*Gal1-GST-ATG31*]	This study
